# Character matters: The network structure of leader character and its relation to follower positive outcomes

**DOI:** 10.1371/journal.pone.0255940

**Published:** 2021-09-01

**Authors:** Lucas Monzani, Gerard H. Seijts, Mary M. Crossan

**Affiliations:** Ivey Business School at Western University, London, Ontario, Canada; University of Salento, ITALY

## Abstract

We investigated the relationship between self-ratings of leader character and follower positive outcomes—namely, subjective well-being, resilience, organizational commitment, and work engagement—in a public-sector organization using a time-lagged cross-sectional design involving 188 leader—follower dyads and 22 offices. Our study is an important step forward in the conceptual development of leader character and the application of character to enhance workplace practices. We combined confirmatory factor analysis and network-based analysis to determine the factorial and network structure of leader character. The findings revealed that a model of 11 inter-correlated leader character dimensions fit the data better than a single-factor model. Further, judgment appeared as the most central dimension in a network comprising the 11 character dimensions. Moreover, in a larger network of partial correlations, two ties acted as bridges that link leader character to follower positive outcomes: judgment and drive. Implications for theory and practice are discussed.

## Introduction

Leader character has emerged as an important underpinning to leadership theories, focusing on who the leader is, which then informs what they do or can do [1]. Hannah and Jennings emphasized that “leaders must have both character and competence and that either by itself is deficient” [2, p. 42]. Similarly, Hannah and Avolio stated that “character is an indispensable component of leadership and its development” [1, p. 979]. Yet, as Hannah and Avolio describe, “Most current leadership theories such as leader member exchange or ethical leadership do not include an in-depth discussion of character or other locus that drives such leadership. Moreover, the survey measures associated with these theories tend to focus more on the transmission of leadership than on its source or locus” [1, p. 980]. Likewise, Sturm, Vera, and Crossan pointed out that “whereas the micro- and macro-oriented leadership literatures have often studied leader competencies necessary for effective performance, the role of leader character in relation to competencies and performance has been to a large extent neglected” [3, p. 349]. We aim to fill this gap by exploring how leader character relates to positive outcomes for organizational members, or followers, and the organization as whole.

Although significant progress has been made in identifying what leader character is and why it matters, there are few empirical examinations, perhaps because leader character is a complex construct that has challenged researchers methodologically. We employ a definition of character anchored in the virtue ethics perspective and described by Crossan, Byrne, Seijts, Reno, Monzani, and Gandz as a set of interconnected virtues or dimensions that are manifested “in habits of cognition, emotion and behavior that embody human excellence and produce social betterment” [4, p. 2]. Whereas there is some evidence relating particular dimensions of leader character to individual, team, and organizational performance [5–9], studies tend to treat the dimensions as discrete and often examine dimensions of character in isolation (e.g., humility) [10, 11]. There are no studies linking the interconnected set of leader character dimensions to performance or other organizational relevant outcomes. Our study is the first empirical examination of leader character to address the complex and interconnected nature of the construct and how it contributes to follower positive outcomes. Examining the interconnected nature of character is essential since the ontology of character reveals that what might be a virtuous dimension of character could actually operate like a vice when not supported by the other dimensions. Thus, we address the following research question: How do the interconnected dimensions of leader character influence positive follower outcomes?

The positive follower outcomes we examine are subjective well-being, resilience, organizational commitment, and work engagement in a public-sector organization using a time-lagged cross-sectional design involving 188 leader—follower dyads and 22 offices. Our study is an important step forward in the conceptual development of leader character and the application of character to workplace practices.

In the next sections, we first provide a brief overview of existing theory and research on virtuous character, including the leader character framework we employ, and present our hypotheses. We then describe the methodology we used to test our hypotheses. Finally, we discuss the results, highlight the theoretical and practical significance of our findings, and offer opportunities for future research.

### Theoretical background

Consistent with the definition of character we provided earlier, many leader character scholars anchor their discussion of character in virtuous character [1, 4, 12–17]. For example, building on the seminal work of Peterson and Seligman [18], Crossan et al. conceptualized character as an amalgam of virtues, values, and personality traits that enable excellence [4].

Virtues are contextually appropriate behaviors that are widely considered as emblematic of good leadership (e.g., integrity, courage, humility, and temperance). A few of these virtuous behaviors reflect the activation of personality traits, such as resiliency, conscientiousness, and openness to experience, which are relatively stable dispositional variables [19, 20]. Such personality traits predispose individuals to behave in certain virtuous ways if not overridden by contextual variables such as job design, incentive systems, or peer pressure. And a few of the virtuous behaviors express values, such as transparency and acting with candor. Values are an acquired set of deep-seated beliefs about what is morally right or wrong, and they remain relatively stable across contexts and situations [21, 22]. While all of the behaviors associated with character are virtuous, most behaviors are neither personality traits nor values. Thus, character can be developed [23–25].

**Fig 1:**
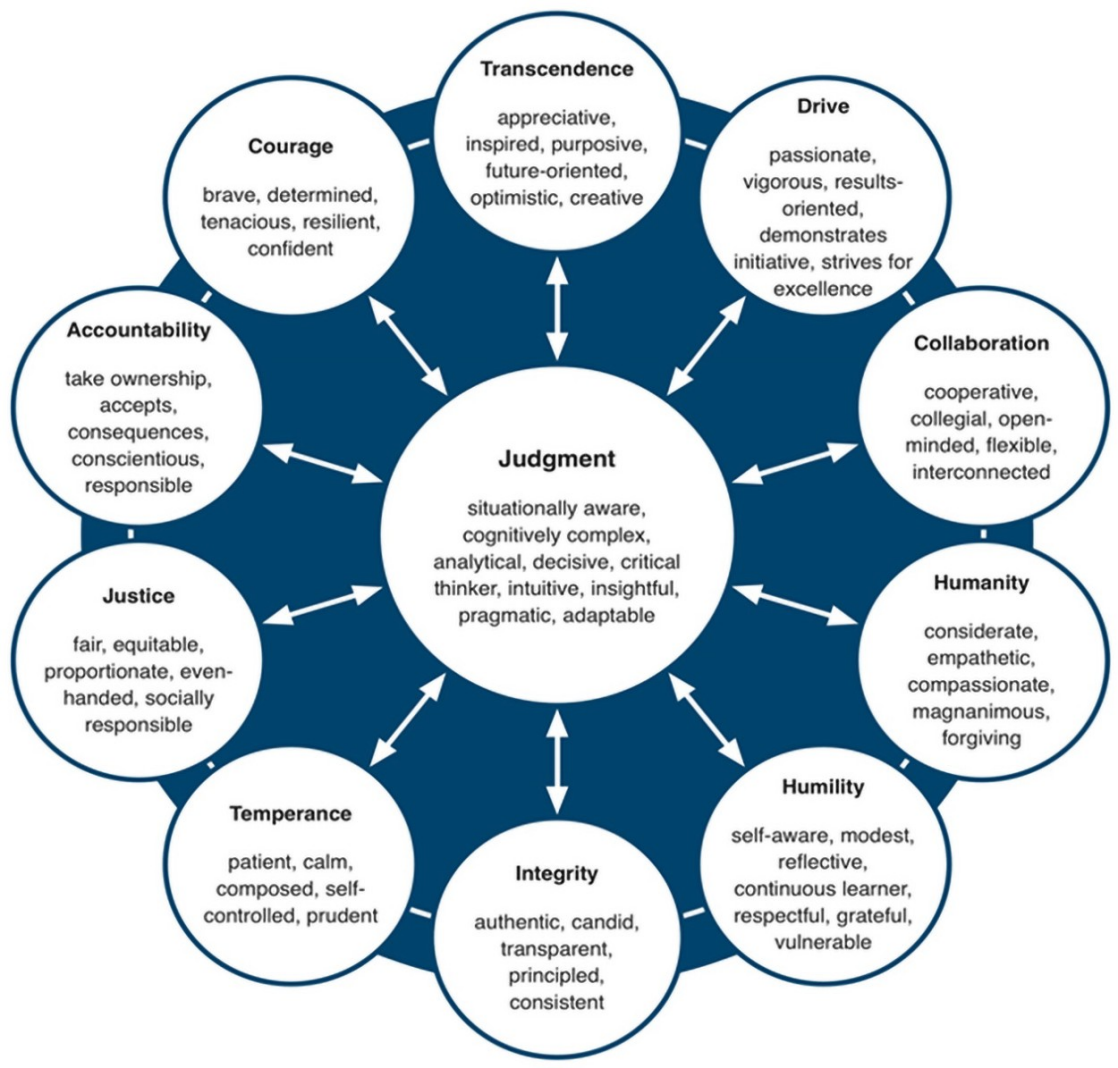
Character dimensions and associated character elements.

In our study, we adopted the leader character framework developed and validated by Crossan and colleages [4]. They invoked an engaged scholarship approach in developing a framework of character that applies to leadership in organizations. Their series of qualitative and quantitative studies involving over 2,500 leaders from the public, private, and not-for-profit sectors led to the identification of 11 character dimensions and 61 character elements that were widely considered by leaders to be emblematic of good leadership (Fig 1). Crossan and her colleagues use the term “character dimension” to refer to latent constructs and the term “character elements” to refer to the virtuous behavioral indicators that illustrate the latent construct. Several of these behavioral indicators include a cognitive or emotional component, which is consistent with the definition of character provided earlier. For example, the dimension of judgment implies that individuals make sound decisions in a timely manner based on relevant information and critical analysis of facts, often in uncertain, complex, and ambiguous circumstances. These behaviors have a strong cognitive aspect. Similarly, the dimension of humanity implies that individuals appreciate and identify with others’ values, feelings, and beliefs. These behaviors have a strong emotional aspect.

There are two core foundations to the Crossan and colleages’ leader character framework [4]. First, the positioning of judgment in the center of the framework is wholly consistent with Aristotelian thinking. Aristotle contended that practical wisdom—or judgment—acts as a self-regulatory mechanism that enables the enactment of the virtues in situationally appropriate ways. For example, the wise leader embodies strength in the dimensions of character that enable the leader to both understand and express when it is appropriate to demonstrate drive and when to be patient and considerate of other people’s concerns; when to demonstrate humility and when to be assertive; and so forth. Current frameworks of leader character recognize judgment as a dimension of character but have not empirically accounted for the central role judgment plays to connect the other character dimensions [15, 18].

Second, the inter-connectivity between the dimensions of character and their corresponding elements is crucially important. This is because what could be virtues may actually operate as vices when not supported or accompanied by other dimensions of character [4, 26]. For example, courage may manifest itself as recklessness when not supported by temperance, which affords patience and emotional self-regulation. This observation may explain deficiencies in judgment by leaders who demonstrate behaviors associated with, for example, drive, yet do so in a way that reveals little compassion and justice. Hence, the framework provides an important caution to both scholars and practitioners who focus on only one dimension, such as courage, drive, or integrity, in explaining or predicting leader effectiveness. This consideration is particularly important given the “too much of a good thing” phenomenon, which has recently been discussed and documented in both the psychological [27, 28] and management [29, 30] literatures. For example, many managerial behaviors that are traditionally seen as having a positive and linear relationship with performance are being revisited with a more critical lens. Studies have shown that behaviors associated with individual difference variables such as assertiveness [31] and conscientiousness [32] have curvilinear relationships with leadership in multiple samples and subjective and objective measures. When treated in isolation, studies do not address the possibility that high levels of conscientiousness (accountability) need high levels of temperance to avoid burnout, for example, nor the necessary role of judgment, through behaviors such as situational awareness and being adaptable, to ensure that what could be virtuous behavior does not manifest as a vice in any given situation.

#### Operationalizing leader character

The ontological nature of character should guide its operationalization. While empirical studies of leader character are relatively rare, those that examine character tend to treat it like a personality construct or, at best, as an unconnected collection of dispositional variables with two primary conceptual and methodological shortcomings. The first shortcoming is that only parts of leader character are examined and hence it is not possible to examine whether what might be a virtue is actually operating as a vice. Prior studies have tended to explore the effect of a single character dimension on an outcome of interest without considering that such effects might be informed by other character dimensions. For example, researchers may consider managerial efforts to foster voice to be an issue of integrity and, in particular, the need to openly discuss and condemn wrongdoing when it occurs in the organization. Yet, at the same time, to be constructive, such managerial efforts require the simultaneous activation of other leader character dimensions, including, but not limited to, courage (to challenge organizational members on their dysfunctional behaviors), collaboration (to avoid the act of speaking up ending in a lonely struggle to rectify the wrongdoing), temperance (to develop the patience that is necessary to influence and persuade important stakeholders), and humility (to avoid individuals interpreting the actions of the person exercising voice as sheer arrogance or inspired by self-interest).

The second shortcoming is that even when multiple character dimensions are examined, the methodologies employed do not account for the dimensions of character as interconnected. Rather, techniques such as factor analysis are used to tease apart dimensions. This approach is inconsistent with the underlying ontology of character and risks shifting the study of character toward extracting aspects of character that do not fit neatly into unidimensional or shared variance approaches. For example, Riggio et al. created the Leadership Virtues Questionnaire, which assesses four virtues: prudence, fortitude, temperance, and justice [15]. The results of both exploratory and confirmatory factor analyses revealed that a single-factor model best captured a construct that Riggio et al. labeled ethical leadership. Using hierarchical linear regression, they then reported that ethical leadership had a positive effect on psychological empowerment, organizational identification, and moral identity of followers. This analytical strategy does not fit with the theorizing of leader character as a set of interconnected virtues or dimensions that collectively determine decision-making and subsequent action; instead, the strategy focuses on what the four virtues have in common, namely, ethical leadership.

The theoretical foundation of leader character is based on the notion that there are 11 unique dimensions of character that—independently and interactively—influence decision-making. Hence, it is critically important that the operationalization of leader character is consistent with the underlying theory. Therefore, we tested two possible and competing approaches. First, consistent with Crossan et al., character is operationalized as 11 dimensions that are distinct and intercorrelated [4]. Second, character is operationalized as a single unidimensional construct. Our first hypothesis is as follows:

Hypothesis 1: A model including 11 inter-correlated character dimensions captures the ontology of character better than 11 inter-correlated dimensions or a single-factor, unidimensional model.

#### Operationalizing leader character as a network

Recent advances in network theory and psychometric research now enable researchers to test leader character as a network by applying the insights from both Exponential Random Graph Models [33] and generalized psychometric networks [34]. The use of network-based analyses is gaining traction in both the behavioral sciences [35, 36] and management [37, 38] literatures because the approach enables researchers to address the methodological challenges that arise from studying highly interconnected constructs. Hence, instead of studying a collection of leadership constructs in isolation, we posit that strength of leader character is revealed not only in the strength of each character dimension but also in their connectedness, thus addressing the potential for a virtue to operate as a vice, as described previously.

While Crossan and colleagues advocated for network-based analysis to investigate the inter-connectivity between the dimensions and elements of leader character, they did not use statistical analysis to test whether the likelihood that judgment occupied a central position within the network occurred by chance [4]. We seek to empirically test whether judgment is the most central dimension in the leader character network by using exponential random graph models to test statistically whether the betweenness centrality index of judgment is higher than those of the other dimensions in the leader character network. Our second hypothesis is as follows:

Hypothesis 2: Judgment is the most central dimension in the network of 11 inter-correlated leader character dimensions.

#### Leader character and follower positive outcomes

Our literature review suggests that there are no studies linking the interconnected set of leader character dimensions (and their associated elements) to performance or other individually and organizationally relevant outcomes. Therefore, to our best knowledge, this is the first empirical examination of character that addresses the complex and interconnected nature of character and how it contributes to follower positive outcomes.

*Self-oriented follower positive outcomes*. We understand self-oriented positive outcomes as those individual psychological states that reveal an optimal level of individual functioning. For example, subjective well-being is a positive psychological state described by positive affect (emotional well-being), a feeling of meaning and purpose in life (psychological well-being), and the belief that one’s actions contribute in a positive way to society as a whole (social well-being) [39, 40]. Subjective well-being should also matter for organizational leaders because it has been related to several employee-related outcomes, including better interpersonal behaviors, higher trust and work engagement, lower turnover, and superior work performance [41–43].

Positive leadership behaviors have been linked to follower well-being [44], whereas such behaviors also attenuate toxic outcomes experienced by followers, such as burnout and distress [45]. For example, meta-analytic findings showed a negative effect of authentic leadership behaviors on follower toxic outcomes, such as burnout including emotional exhaustion [46]. A longitudinal study revealed that the negative effect of authentic leadership on emotional exhaustion was mediated by procedural justice [47]. When leaders display self-awareness, a component of authentic leadership, they become more mindful of or attentive to how fair and equitable behaviors impact others. The ability to self-regulate such behaviors protect follower well-being. Behaviors that are reflective of transformational leadership have also been reported to have a positive effect on well-being. For example, displaying behaviors such as individual consideration may signal to followers that their leaders are concerned with their well-being [48].

A second psychological state that supports individual optimal functioning is resilience, often described as the ability to “bounce back” and endure stressful situations [49, 50]. In the aftermath of the global recession, Robertson, Cooper, Sarkar, and Curran wrote that “the need for personal resilience, especially in the workplace, has never been greater” [50, p. 534]. Meta-analytic and systematic reviews of employee resiliency have shown that resiliency is a predictor of both in-role and extra-role performance [51]. Further, resiliency has a strong, positive effect on mental health and subjective well-being [50, 52].

Research has shown that empowering leadership behaviors (e.g., enhancing the meaningfulness of work, fostering participation in decision-making, expressing confidence in high performance, and providing autonomy from bureaucratic constraints), proactive personality, and optimism are significantly related to resilient behaviors [53]. Further, Gaddy, Gonzalez, Lathan, and Graham [54] found a positive relation between the perception of authentic leadership behaviors and subordinate resilience. Martínez-Martí and Ruch [55] found that character strengths had a predictive value over and above other resilience-related factors, such as positive affect, self-efficacy, optimism, social support, self-esteem, and life satisfaction, as well as sociodemographic variables.

However, as we stated earlier, a limitation of the existing body of research on leader character is that empirical studies do not reflect the holistic perspective that character presents. Instead, researchers tend to examine the effect of leader character dimensions and elements on variables of interest in isolation. Or, put differently, studies tend to focus on a particular phenomenon of interest without situating it within the more comprehensive ontology of character. This is problematic because an excess of determination and confidence displayed by a leader may be interpreted by followers as arrogance or insensitivity in the absence of empathy and compassion, thereby undermining well-being and resilience. Similarly, it is hard to envision how the dimension of integrity can stand on its own to facilitate subjective well-being and resilience. This is because integrity without collaboration and humanity may become dogmatic and rigid, hence damaging subjective well-being and resilience.

*Task-oriented and organization-oriented follower positive outcomes*. A psychological state related to optimal task functioning is organizational commitment, which refers to an individual’s psychological attachment to an organization [56, 57]. There is substantial evidence that having a strongly committed workforce leads to positive outcomes for organizations. For example, organizational commitment has been related to several work outcomes, including lower turnover, absenteeism, and stress, as well as higher attendance, organizational citizenship behaviors, and job performance [58, 59]. As such, organizational commitment is an important measure for leaders to attend to and develop in the people who work for the organization.

Leadership behaviors have been shown to be a contributing factor in the development of organizational commitment. It seems highly plausible that several of the leader character dimensions—accountability, collaboration, drive, humility, and integrity—contribute to expressions of organizational commitment on the part of employees and, eventually, contribute to sustained organizational excellence. For example, Jackson, Meyer, and Wang found that behavioral categories associated with transformational leadership, such as transcendence, collaboration, and humanity, were positively associated with organizational commitment [60]. Further, fairness, which is an important component of transformational leadership, was associated with organizational commitment. Studies have also shown that behaviors that reveal leader authenticity, such as relational transparency and self-awareness, predict organizational commitment [61, 62].

A second psychological state that matters for organizations is work engagement. Work engagement has been described as a psychological state of full presence while conducting one’s work-related tasks [63, 64]. It is characterized by immersing oneself in a task with vigor, absorption, and dedication. Work engagement is a strong predictor of task performance and discretionary organizational behaviors [65] and a negative predictor of burnout [66].

Research has documented the positive effect of certain positive leadership behaviors—including, but not limited to, authenticity, humility, and fairness—on work engagement [46, 67]. For example, Tims, Bakker, and Xanthopoulou [68] found that the daily display of transformational leadership behaviors such as inspirational motivation and individual consideration related positively to employees’ daily work engagement. Babcock-Roberson and Strickland [69] reported that work engagement mediated the relationship between charismatic leadership (emphasizing leadership behaviors such as demonstrating ethical conduct and a clear sense of purpose as well as exhibiting confidence and taking risks) and organizational citizenship behaviors.

However, it is difficult to develop a full appreciation for the effect of leader character dimensions (and associated behaviors) on task-oriented and organization-oriented follower outcomes in the absence of considering the connectedness to other dimensions. Collaboration without justice, for example, may lead to friction between leaders and employees, thereby not only making the implementation of strategy more challenging but also undermining work engagement. Similarly, drive without transcendence may result in ideas or activities that lack focus, contribute to frustration, and eventually hurt organizational commitment. For this reason, we need to consider how the interconnected set of dimensions of leader character influence follower outcomes.

Some evidence points to the merits of examining the influence of interconnected dimensions of leader character on work-related attitudes, including organizational commitment and work engagement. For example, Sousa and Van Dierendonck examined the effect of humility, the backbone of servant leadership, on work engagement [67]. However, they also explained that servant leadership is a balancing act between a humble attitude of service and behaviors that are action-driven, akin to drive. Their results showed a three-way interaction between a humble service attitude, action orientation, and the position of the leader in the organizational hierarchy on work engagement. This result led Sousa and Van Dierendonck to conclude that their study “comes to demonstrate the importance of incorporating additional contingency variables in the further study of servant leadership and the specific mechanisms through which it can affect performance” [66, p. 22]. We follow their lead and aim to answer the question of how leader character, understood as the locus of virtuous leader behavior, affects follower outcomes. Our third hypothesis is as follows:

Hypothesis 3: The leader character network connects to followers’ (a) subjective well-being, (b) resilience, (c) organizational commitment, and (d) work engagement, conforming a single larger network.

## Materials and methods

### Participants

Our sample consists of 66 office managers and 421 direct reports from a large Canadian public organization. After discarding cases due to missing data, managers and their respective reports were matched into 188 leader—follower dyads. The office manager sample consisted of 40 men and 25 women; 1 participant did not indicate their gender. The direct report sample consisted of 124 men and 185 women; 112 participants chose not to disclose their gender. No other demographic data was collected at the request of the organization. Participants responded to an e-mail invitation from the researchers to participate in a survey on leader character and well-being. Participation in the survey was voluntary. The response rate was 53% for the office managers and 29% for the direct reports. Our anonymized datasets are publicly available at: https://osf.io/v23ak/?view_only=9dd7aba566774bdd8f6f21f48524ff6d.

### Procedure

Our study was approved by Research Ethics Board of Western University (NMREB File # 105963). As per the research ethics guidelines of our university, (a) all participants provided their informed consent to participate in our study, (b) participation was completely anonymous and voluntary, (c) and participants provided their informed consent electronically by clicking in a button with the following text: “I provide my informed consent to participate in this survey”. None of our participant declined to participate nor withdraw of the study once started.

After providing their informed consent, participants completed an online survey. The office managers completed a self-report measure of leader character. The direct reports completed ratings of subjective well-being, resilience, organizational commitment, and work engagement. The time lag between these two measurements was six months. The ratings were matched through a unique code.

### Measures

#### Leader character

We used the leadership character insight assessment developed and validated by Crossan and her colleagues to measure leader character [70]. Office managers were asked to rate the likelihood they are able to demonstrate 61 leader-specific behaviors. These behaviors can be classified into 11 leader character dimensions: accountability, collaboration, courage, drive, humanity, humility, integrity, judgment, justice, temperance, and transcendence. A sample item for the leader character dimension of courage is “Displays resolve and stays committed to see things through.” The scale scores ranged from 1 (extremely unlikely) to 5 (extremely likely); the midpoint was 3 (unsure).

#### Subjective well-being

We used the 14-item short-form of the Mental Health Continuum [40] to measure subjective well-being. The scale has three dimensions: emotional, social, and psychological well-being. The scale has good validity and reliability [71]. A sample item is “How frequently do you feel that you have experiences that challenge you to grow and become a better person?” The scale scores ranged from 1 (not true at all) to 5 (frequently, if not always); the midpoint was 3 (sometimes).

#### Resilience

We used a 10-item version of the Connor-Davidson resilience scale to measure resilience [72]. The scale has two dimensions: hardiness and persistence. The scale has good validity and reliability [73]. A sample item is “I tend to bounce back after illness or hardship.” The scale scores ranged from 1 (not true at all) to 5 (true nearly all of the time); the midpoint was 3 (sometimes true).

#### Organizational commitment

We used the 9-item reduced version of Meyer and Allen’s [56] organizational commitment scale to assess commitment to the organization. The short-form scale captures the three types of commitment: affective, normative, and continuance commitment. The scale has good validity and reliability [74]. A sample item is “This organization has a great deal of personal meaning for me.” The scale scores ranged from 1 (strongly disagree) to 5 (strongly agree); the midpoint was 3 (neither agree nor disagree).

#### Work engagement

We used the short-form of the Utrecht work engagement scale [75] to measure work engagement. The scale consists of nine items, loading on three dimensions: vigor, absorption, and dedication. The scale has good validity and reliability [64]. A sample item is “I am enthusiastic about my job.” The scale scores ranged from 1 (never) to 7 (every day); the midpoint was 3 (sometimes).

### Data analysis

The data analysis proceeds in four steps. First, to test our Hypothesis 1, we conducted a confirmatory factor analysis (CFA) to determine the factorial structure of the leader character measure. Second, we constructed a network using each character dimension as a node and using the character dimensions’ partial correlation coefficients with the other dimensions as connecting ties. Third, to test Hypothesis 2, we calculated the resulting network’s descriptive indicators and examined whether judgment emerged as the most central dimension using the betweenness centrality index [76]. We used Exponential Random Graph Modeling (ERGM) to assess whether the observed network of leader character dimensions simply occurred by chance (p >.05, a random model) or the probability of observing this network by chance is smaller than.05. We also tested whether including the betweenness centrality index of each character dimension as predictors of the network structure significantly increased the odds that the observed network did not occur by chance. A p value < .05 for the betweenness centrality index indicates such network structure did not occur by chance. Fourth, to test Hypothesis 3, we employed a tie selection algorithm. The algorithm enables us to test whether the network of leader character dimensions connects with the measures of subjective well-being, resilience, organizational commitment, and work engagement. Hypothesis 3 is confirmed if, after running the threshold algorithm, the partial correlation matrix of the leader character dimensions and the follower outcomes conform a large, single network. Hypothesis 3 would receive further support if the follow-up ERGM regressions show that such network structure does not occur by chance.

#### Confirmatory factor analysis

We conducted a CFA to examine whether the 11-dimension model of leader character proposed by Crossan et al. fits our data well [4]. We used MPLUS 7.0 to conduct the CFA [77]. Since our dataset possesses a relatively low N for the number of parameters in our model (N/parameter ratio), we used a robust indicator, namely, the Weighted Least Squares—Mean and Variance Adjusted (WLSMV). The WLSMV estimator provides a good recovery of population parameters even when the ratio of the number of observations to the variables is below ten (N/p < 10) or the number of parameters in a model is above five (N/q *>* 5). For example, based on a Monte-Carlo simulation, Moshagen and Musch suggest that samples as small as N = 50 are often sufficient to obtain accurate factor loadings [78].

In addition, we calculated the χ2/df ratio to compare parsimony between models and evaluate differences in practical fit indices. We took the χ2, χ2/df ratio, Root Mean Square Error of Approximation (RMSEA), Tucker’s Fit Index (TFI), and the Comparative Fit Index (CFI) as the goodness-of-fit indicators [79]. Although there are no generally accepted fit standards, some criteria have been proposed to interpret differences in practical fit indices. For example, Widaman (1985) argued that differences smaller than 0.01 in TFI values associated with models is an indication of negligible differences. Cheung and Rensvold [79] suggested that decreases in fit for CFI values greater than 0.01 might be meaningful. Finally, Chen suggested that when the RMSEA increases by less than.015, then the difference in fit index is not meaningfully different. RMSEA values below.05 and TFI and CFI values between.90 and.95 would indicate an acceptable fit for our CFA model [80].

#### Network-based analyses

Network theory is a proper approach to ascertain the interconnections between complex, correlated constructs in management research [38]. One area in network theory that has attracted a lot of attention is generalized network psychometrics or GNP [34]. GNP is an application of network theory to psychometrics that focuses on the structural configuration of related constructs—that is, their topology. Instead of inverting the psi-matrix (the matrix of correlations between latent constructs) into a gamma matrix, GNP uses the inverse of the psi-matrix to estimate parameters and derive partial correlations. Given that a partial correlation is the remaining effect between two variables after removing the spurious effects from all the variables in a system (that is, the network of correlations), its use contributes to reducing the potential endogeneity between latent constructs—a significant concern in leadership studies [81].

GNP relies on a more efficient estimation approach, the gamma shrinkage estimator, for parameter estimations. This procedure allows researchers to test for relationships among variables with a much lower N/Parameter ratio than structural equation models (SEM) models require. In short, a shrinkage covariance estimator is an improved covariance estimator that has a guaranteed minimum mean squared error, is well-conditioned, and provides a positive definite psi-matrix, even for small sample sizes. The shrinkage covariance estimator was developed to address the problem of inferring a large number of large-scale associations in networks with a relatively low number of observations, also known as the “small *n*, large *p*” problem in genomic research. (For a detailed explanation and a mathematical demonstration, see [82]).

We used R v.3.40 statistical software for all network-based analyses, including the packages Statnet [83], Igraph [84], and GeneNet [85]. There are at least two advantages of adopting a network-based analytical approach to understand leader character. First, the leader character framework proposed by Crossan and colleagues assumes that the character dimensions and their elements are interconnected and that judgment is the most central dimension in the network [4]. Unfortunately, CFA does not allow us to draw topological inferences regarding the relationships between latent constructs such as the leader character dimensions. For example, whereas CFA decomposes a covariance matrix to determine which character elements load onto the respective character dimensions, CFA does not allow us to determine which dimension or latent construct is most central. Hence, extending CFA analysis with a network-based analytical approach is required.

The second advantage of a network-based analytical approach involves the modeling of the data. For example, in CFA analyses, when dealing with latent constructs that are highly correlated, a main issue for data modeling software packages is the resulting multi-collinearity, which is likely to impede the inversion of the psi-matrix and thus create negative residual variances. Instead, GNP allows for nodes (in our case, leader character dimensions) to be highly correlated; hence, it is a method that fits our theorizing on leader character. We selected both the Statnet and GeneNet packages because they are a proper alternative in handling multi-collinearity in latent constructs and give us more flexibility in our data modeling efforts than other statistical packages given our relative low N/parameter ratio.

*Network construction*. Our study attempts to extend the network-based analytical approach employed by Crossan et al. in two distinct ways [4]. First, Crossan et al. used the Pearson’s product-moment correlation (r) as ties to connect the nodes at their item-level and construct-level networks [4]. This procedure might result in spurious correlations between the leader character elements and/or dimensions. We used partial correlations as ties instead. We employed the aforementioned GeneNet statistical package to calculate the network of partial correlations for leader character elements and dimensions. GeneNet uses a statistical inference approach to decide which ties are to be included in the final network by estimating the values of all the pairwise partial correlations between the network’s variables after shrinkage estimation correction was applied.

Second, we developed a threshold detection procedure to minimize researcher bias in the construction of the item-level and construct-level networks. A critical decision in constructing a network is defining which threshold will determine whether a tie should be included in the network’s adjacency matrix—that is, the matrix that involves all significant partial correlations across nodes. Because choosing an adjacency threshold implies a subjective decision for the researcher, such a decision is susceptible to potential confirmation bias. To prevent such potential bias, we adopted a computational approach to determine the adjacency threshold. We instructed the R software to establish as a threshold the partial correlation value of the lowest tie that was significant at the p < .05 level. This approach optimizes the number of ties included in favor of the more parsimonious network.

*Descriptive analysis of network-based data*: *Centrality*. Researchers can rely on the topological properties of a given network to describe its unique structure and mechanisms at play. For example, researchers can identify which node connects other nodes best as a result of its central position within the network (the betweenness centrality index) [76]. Such descriptive statistics can be used to make statistical inferences about the structure of a network. The betweenness centrality index is the best indicator to test Hypothesis 2. The importance of occupying a central position within a network is derived from the node’s ability to connect other nodes more efficiently than other, more peripheral nodes could [86]. However, to test whether the leader character network does not occur by chance, and whether a node’s betweenness centrality index explains deviation from a random model, we need to rely on ERGMs.

To test Hypotheses 2 and 3, we used ERGMs, which are the network analysis equivalent to logistic regression models. The output of an ERGM analysis indicates the probability that an observed network did not occur by chance. Further, researchers can enter additional equation terms into the ERGM algorithm to test the unique contribution of specific predictors (e.g., specific node attributes or a dyadic property between two nodes) and hence test more nuanced models. For example, homophily is a dyadic property that refers to the tendency of a node to form ties with nodes that share a common characteristic (e.g., each character dimension is more likely to form ties with other character dimensions rather than with individually or organizationally relevant variables such as subjective well-being and organizational commitment).

The first step in an ERGM analysis is to construct a baseline network model. To do this, researchers must compare the observed network model against a set of randomly generated network models with the same number of nodes (in our case leader character elements), using a computational approach similar to a Monte-Carlo simulation. The baseline network model can be expressed as a single-term ERGM equation with one predictor, namely, the existence of ties between nodes. This equation term is the ERGM equivalent to the constant term in a logistic regression analysis. The contrast and interpretation of baseline ERGM models share the same logic behind analyses based on maximum likelihood and χ^2^ distributions, such as SEM. ERGM models use a log-likelihood (LL) deviance approach to compare how much a baseline network model differs from a set of randomly generated networks.

The second step in an ERGM analysis, again analogous to a logistic regression analysis, is to compare more complex models against the baseline model by entering additional terms into the ERGM equation. This allows the researcher to determine to what extent each new equation term (or predictor) explains how well more nuanced models fit the data. Thus, in our analysis, a second equation term includes predictors exploring main effects (e.g., whether a node is a leader character dimension or a dimension of subjective well-being or work engagement), and a third equation term includes interactive effects (e.g., dyadic properties such as homophily). This stepwise approach allows the researcher to isolate the unique contribution of each predictor toward the overall deviance (-2LL) from the baseline model [87].

The third and final step is to test the model’s fit. Similar to SEMs, ERGM equations require the use of goodness-of-fit indicators. The overall deviation between the randomly generated, null model and the observed baseline network models is captured by the LL statistic. The LL statistic is analogous to the residual sum of squares in multiple regression, indicating how much unexplained information remains after fitting the model to the data. Large values of the LL statistic indicate poorly fitting models. Further, most ERGM software packages such as Statnet use the LL statistic to calculate both the Aikake (AIC) and the Bayesian information criterion (BIC) for each model. The AIC and BIC are two well-established goodness-of-fit indicators for non-nested models. Although there are no cut-off points for the AIC and the BIC, lower scores indicate a better model fit.

To test Hypotheses 3, we used an ERGM to determine whether the baseline model for the partial correlation network connecting the leader character dimensions to the variables of interest—subjective well-being, resilience, organizational commitment, and work engagement—deviated significantly from a random or null model. We then included several predictors in two ERGM equations. In the first equation, we took the character dimensions and the aforementioned variables as predictors, using the subjective well-being dimensions as the reference group (as required by a log-likelihood approach). We added homophily as an interaction term in the second equation. This procedure helps to isolate the unique contribution of each construct to explain the overall network structure [88].

## Results and discussion

### Descriptive statistics

The means and standard deviations of the variables measured are shown in Table 1. Table 1 also reports the Pearson correlation coefficients among the variables.

**Table 1:** Descriptive statistics and Pearson’s bivariate correlations for all measured constructs (N = 188).

	M	SD	**1**.	**2**.	**3**.	**4**.	**5**.	**6**.	**7**.	**8**.	**9**.	**10**.	**11**.
**1. L-Jud**	4.45	.36	(.88)										
**2. L-Tra**	4.22	.50	.51**	(.72)									
**3. L-Dri**	4.34	.47	.46**	.55**	(.81)								
**4. L-Col**	4.62	.33	.28**	.47**	.52**	(.68)							
**5. L-Hua**	4.59	.36	.24**	.28**	.22**	.55**	(.83)						
**6. L-Hum**	4.55	.39	.49**	.53**	.63**	.48**	.44**	(.85)					
**7. L-Int**	4.60	.29	.55**	.26**	.37**	.33**	.48**	.59**	(.66)				
**8. L-Tem**	4.13	.53	.51**	.31**	.29**	.20**	.28**	.07	.48**	(.84)			
**9. L-Jus**	4.52	.41	.64**	.69**	.53**	.48**	.44**	.53**	.52**	.53**	(.82)		
**10. L-Acc**	4.74	.27	.47**	.32**	.45**	.14**	.33**	.23**	.24**	.38**	.49**	(.72)	
**11. L-Cou**	4.58	.29	.57**	.28**	.59**	.40**	.44**	.65**	.74**	.38**	.49**	.35**	(.60)
**12. F-PWB**	4.20	.62	.08	.12	.07	.06	.07	.04	.12	.22*	.12	.03	.08
**13. F-EWB**	4.25	.72	.05	.00	.08	-.03	-.03	-.06	-.02	.12	.04	.03	.01
**14. F-SWB**	3.79	.82	.12	.11	.13	.02	.00	-.01	.08	.19**	.07	.08	.06
**15. F-AC**	3.69	.99	-.11	.06	.16*	.03	.02	.05	-.02	-.04	.00	-.05	-.02
**16. F-NC**	3.36	.99	-.06	.04	.09	.03	-.03	-.03	-.12	-.03	-.01	-.04	-.09
**17. F-CC**	3.16	1.02	.15*	.02	.05	.09	.04	.06	.05	.00	.10	.05	.05
**18. F-Vig**	4.97	1.45	-.01	.01	.04	-.09	.00	-.01	-.04	.09	.02	.03	.00
**19. F-Abs**	5.66	1.03	-.02	-.02	-.04	-.01	.11	-.04	.02	.03	.02	.05	-.02
**20. F-Ded**	5.71	1.19	-.02	-.02	-.04	-.11	.03	-.01	.03	.08	-.01	-.02	.01
**21. F-Har**	4.30	.58	.03	.12	.08	.06	-.02	.04	-.02	.04	.08	-.01	.03
**22. F-Per**	4.41	.58	.04	.08	.07	.06	.01	-.04	-.03	.08	.03	-.04	-.06
Cont’d	M	SD	12.	13.	14.	15.	.16	.17	.18	.19	.20	.21	.22
12. F-PWB	4.20	.62	(.86)										
13. F-EWB	4.25	.72	.66**	(.70)									
14. F-SWB	3.79	.82	.73**	.62**	(.86)								
15. F-AC	3.69	.99	.20**	.25**	.29**	(.85)							
16. F-NC	3.36	.99	.20**	.26**	.34**	.68**	(.78)						
17. F-CC	3.16	1.02	-.27**	-.22**	-.27**	-.06	.02	(.82)					
18. F-Vig	4.97	1.45	.29**	.42**	.36**	.42**	.45**	-.19**	(.87)				
19. F-Abs	5.66	1.03	.20**	.29**	.23**	.28**	.29**	-.11*	.51**	(.64)			
20. F-Ded	5.71	1.19	.32**	.44**	.36**	.46**	.46**	-.19**	.76**	.57**	(.84)		
21. F-Har	4.30	.58	.43**	.31**	.31**	.13**	.10*	-.19**	.32**	.29**	.32**	(.79)	
22. F-Per	4.41	.58	.47**	.36**	.36**	.16**	.10*	-.19**	.30**	.27**	.31**	.68**	(.77)

Note:

* p < .05;

** p < .01;

Cronbach alphas are shown in the diagonal; L = Leader; F = Follower; L-Acc = Accountability; L-Col = Collaboration; L-Cou = Courage; L-Dri = Drive; L-Hua = Humanity; L-Hum = Humility; L-Int = Integrity; L-Jud = Judgment; L-Jus = Justice; L-Tem = Temperance; L-Tra = Transcendence; F-PWB = Psychological Well-Being; F-EWB = Emotional Well-Being; F-SWB = Social Well-Being; F-AOC = Affective Commitment; F-NOC = Normative Commitment; F-COC = Continuance Commitment; F-Vig = Vigor; F-Abs = Absorption; F-Ded = Dedication; F-Har = Hardiness; and F-Per = Perseverance.

### Confirmatory factor analysis

We ran two CFA models on the full data set of 66 office managers. The results indicated that the best solution involved a model of 11 distinct and inter-correlated factors (χ^2^ (1540) = 1722.85***; RMSEA = .04, CFI = .95, TLI = .94 and WRMR = 0.97). The model of 11 intercorrelated factors fit our data better than a single construct model in which all 61 items loaded onto a single factor (S-B scaled χ^2^ (1595) = 1990.37***; RMSEA = .06, CFI = .88, TLI = .88 and WRMR = 1.24). However, the psi-matrix of the 11-factor model was not a positive definite due to a high multi-collinearity between the Humility and Collaboration dimensions (ψ Hum—Col = 1.07). To address the multi-collinearity issue, we followed recommendations to model a higher-order factor that captured the shared variance among the latent constructs, which we labeled leader character [89]. The results of the CFA revealed that the revised model had an acceptable fit to the observed data (S-B scaled χ^2^ (1584) = 1809.03***; RMSEA = .05, CFI = .93, TLI = .93 and WRMR = 1.06) and fit the data better than the single construct model. Thus, taken as a whole, our CFA provided partial support for Hypothesis 1.

### Network-based analysis

#### Centrality analysis

**Fig 2:**
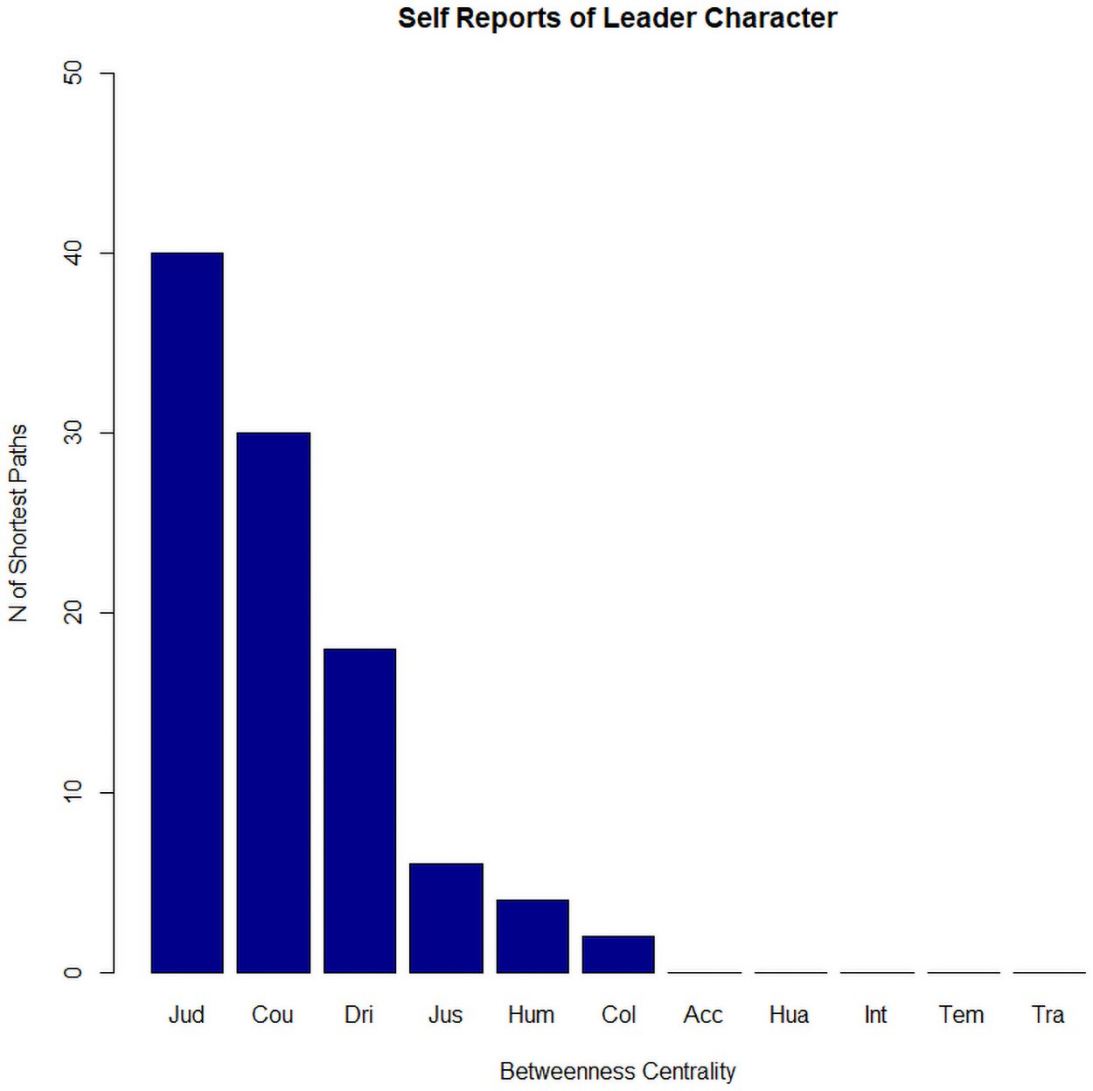
Betweenness centrality of leader character dimensions.

Our centrality analysis revealed that judgment emerged as the most central dimension in the leader character network with a betweenness centrality score of 40. Judgment was followed by Courage with a score of 30, and Drive with a score of 18. The betweenness centrality score of Justice was 6 and the score of Humility was 4. All remaining dimensions showed a score of 0 (See Fig 2).

We then used ERGM to test whether this network configuration occurred by chance. Table 2 shows that the observed baseline network (Model A) deviated significantly from the randomly generated or null network model (Odds Ratio = .57, 95% CI [.45;.73] and Wald’s z = -2.82, p < .001). We then constructed an ERGM equation that includes each dimension’s betweenness centrality index as a predictor (Model B). The results revealed that Model B fit the data better than Model A (Deviance A = -8.30*** vs. Deviance B = -17.90***; ΔBA = -9.60). Further, the betweenness centrality indexes of the 11 leader character dimensions was a significant predictor of the deviance from the baseline model (Odds Ratio = 1.03, 95% CI [1.01, 1.06] and Wald’s z = 2.98 p < .001).

**Table 2:** Exponential random graphs models for the leader character network.

	A: Baseline model	Model B: Main effects
**Network Density**	-.56 (.20)**	-1.23 (.31)***
	OR = .58, [.45,.72] Wald’s z = -2.82*** d = .31, var d = .01	OR = .29, [.16;.54] Wald’s z = -3.93 d = .16, var d = .02
**Node’s BCI**		.03** (.01)
		OR = 1.03, [1.01; 1.06] Wald’s z = 2.98** d = .57, var d = .00
**Null Model**:	Baseline	Main effects
**152.50 (110)**	144.2 (109)	134.6 (108)
**-Δ2LL (df)**	Δ Null-A = 8.29**	ΔA-B = 9.58**
***AIC***	146.2	138.6
***BIC***	148.9	144

Note:

* p < .05;

** p < .01;

*** p < .001;

Density = network density as function of homogeneous edge probability (constant); BCI = betweenness centrality index (main effect); AIC = Akaike Information Criterion; BIC = Bayesian Information Criterion.

**Fig 3:**
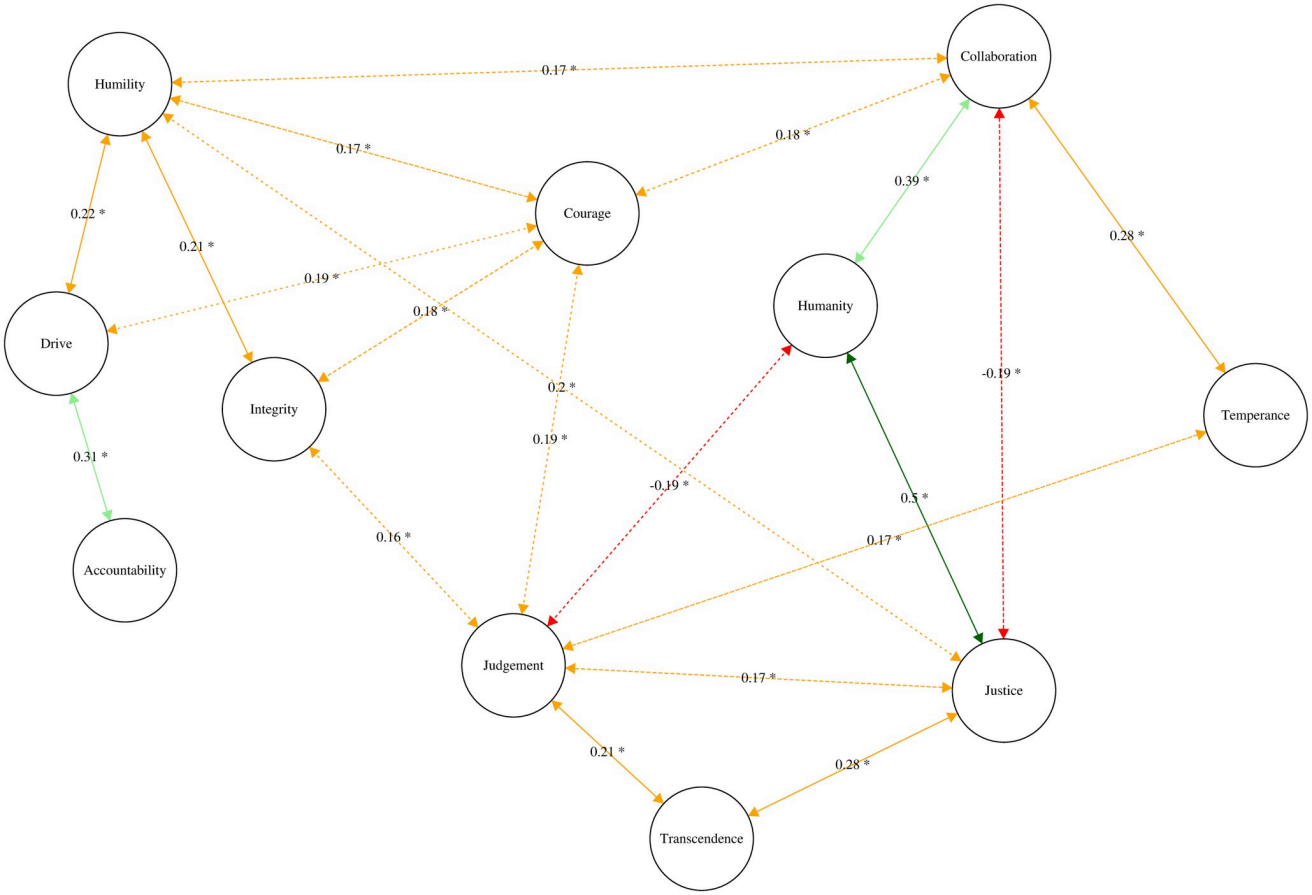
Partial correlation network for leader character dimensions. *Note*: * p < .05.

In sum, the results for the ERGM analysis revealed that judgment emerged as the most central dimension in the network of the 11 leader character dimensions. Hypothesis 2 was therefore supported. Fig 3 illustrates the Leader Character Network in this sample.

#### Extended partial correlation network

**Fig 4:**
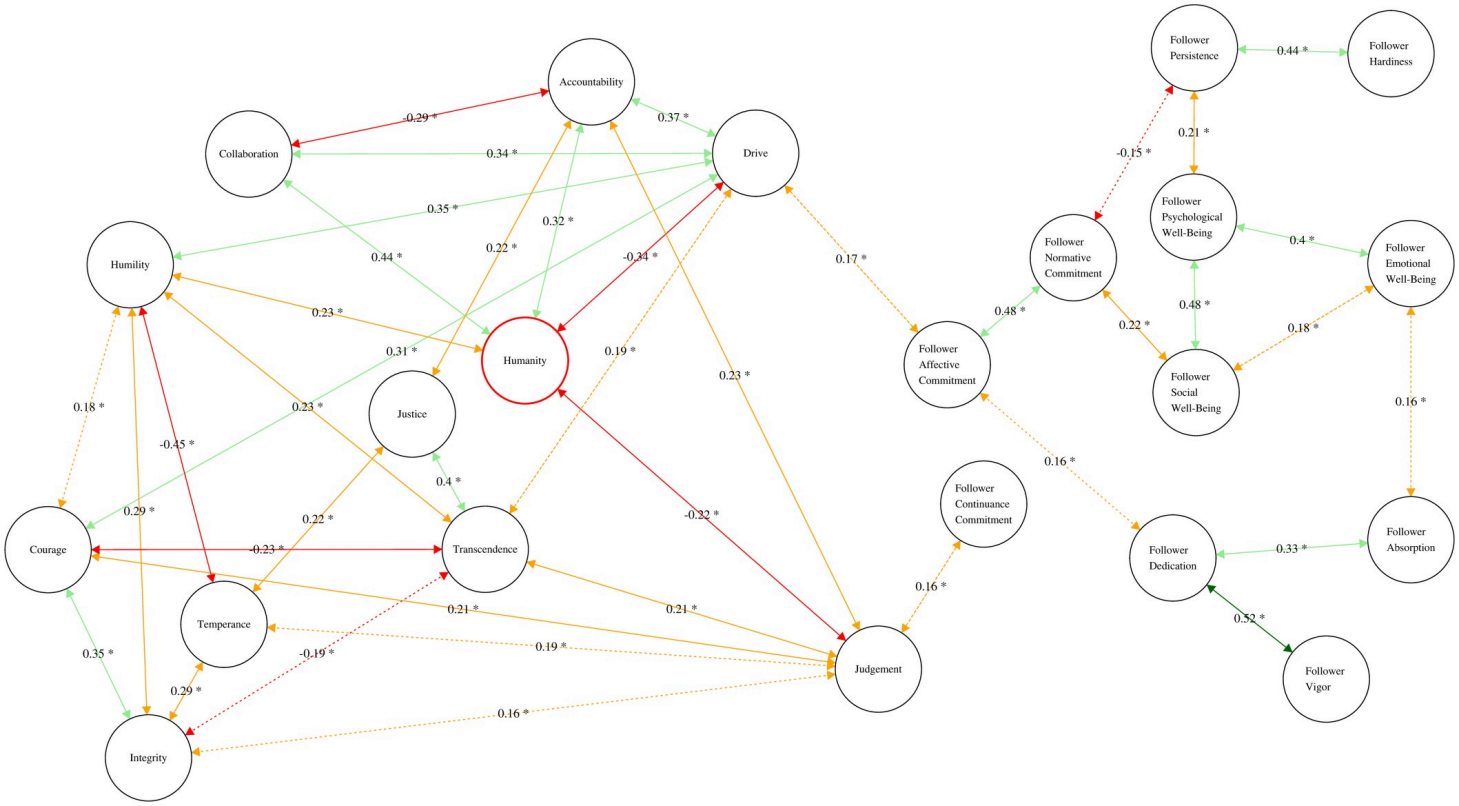
Partial correlation network for leader character dimensions and followers outcomes (dyadic level). *Note*: * p < .05.

Fig 4 shows the most parsimonious partial correlation network, in which the network of leader character dimensions is embedded in a larger network that includes follower subjective well-being, resilience, organizational commitment, and workplace engagement as nodes. This giant component network shows 41 partial correlation coefficients, which are significant at the p < .05 level. Two ties, one connecting leader drive and follower affective commitment (p-r = .17); and one connecting leader judgment and follower continuance commitment (p-r = .16), linked leader character and the four follower outcomes into a comprehensive network.

Table 3 shows three ERGM equations (Models C, D, and E). First, we tested whether the observed or baseline network model (Model C) deviated significantly from a null model. Having established that the baseline model deviated from the randomly generated model, we then explored which properties of the partial correlation network predicted such deviation. Model D included the leader character dimensions and follower outcomes (subjective well-being; resilience; organizational commitment; work engagement) as predictors, taking subjective well-being as the reference group. This means that the three subjective well-being dimensions were set as the base (and coded 0) in the model since ERGM analysis is based on logistic regression.

**Table 3:** Exponential random graphs models for extended network.

	Mode C. Baseline	Model D: Main effects	Model E: Interaction effects
***Network Density***	**-1.53 (.12)**	-2.11 (.51)	-1.89 (.65)
	**OR = .22, [.17,.27]** **Wald’s z = -12.59** *** **;**	OR = .12, [.04;.33] Wald’s z = -4.11**; d = .07, var d = .05	OR = .15, [.04;.54] Wald’s z = -2.89**; d = .08, var d = .08
	**d = .12, var d = .00**		
***Node Attributes***			
**SWB**		Reference	Reference
**LC**		.71 (.30)	-.99 (.41)
		OR = .04, [.28; 1.37] z = 2.46*; d = 1.13, var d = .02	OR = .37, [.17;.84] z = -2.4*; d = .21, var d = .03
**OC**		-.31 (.39) OR = .39, [.34; 1.59] z = -.78 ns; d = .41, var d = .03	.18 (.43) OR = 1.20, [.52; 2.75] z = .42 ns; d = .67, var d = .03
**WE**		-.49 (.41) OR = .62, [.28; 1.37] z = -1.19 ns; d = .34, var d = .03	-.68 (.49) OR = .51, [.19; 1.32] z = -1.40 ns; d = .28, var d = .05
**RES**		-.49 (.46)	-21 (.49)
		OR = .62, [.25; 2.48] z = -1.05; d = .34, var d = .04	OR = 1.00, [.43; 2.32] z = .00; d = .63, var d = .03
***Homophily***			3.73 (.44) OR = 41.66 [17.55, 98.88] z = 3.73***, d = 22.97, var d = .04
Null Model 640.5 (462)	Baseline	Main effects	Interaction effects
	432.20 (461)	401.16 (457)	299.61*** (456)
**-Δ2LL (df)**	Δ Null-C = 218.50***	ΔC-D = 30.87***	ΔD-E = 101.55***
**AIC**	434.00	411.20	311.60
**BIC**	438.20	431.80	336.40

Note:

^†^ p < .10;

* p < .05;

** p < .01;

*** p < .001;

LC = Leader character nodes; WE = Work Engagement; OC = Organizational Commitment; SWB = Subjective Well-being.

The results in Table 3 show that the leader character dimensions predicted deviance (-2LL) from the null model (Δ-2LL = 239.31***) above and beyond the deviance of the baseline model (Δ-2LL = 208.44***). More precisely, there was a main effect for the character dimensions (Odds Ratio = .04, 95% CI [.28; 1.37] and Wald’s z = 2.46*; d = 1.13, Var d = .02) in explaining the overall network configuration.

When we included homophily as an interaction term in Model E, it not only increased the deviance from the null model (Δ-2LL = 340.86***) but also substantially improved the fit to our data as evidenced by the lower AIC (Δ = -99.60) and BIC (Δ = -95.40) scores (see Table 3). The homophily term showed that the tendency of the character dimensions to band together rather than be associated with the four follower outcomes was also a significant predictor of the overall network configuration (Odds Ratio = 41.66, 95% CI [17.55, 98.88] and Wald’s z = 3.73***; d = 22.97, Var d = .04). None of the dimensions for resilience, organizational commitment, and work engagement were significant predictors of the overall network structure in model E. These findings suggest that the leader character dimensions and their grouping were the main driver of the overall network configuration. These results support Hypothesis 3.

## Discussion

We sought to develop a better understanding of the network structure of leader character and its influence on follower positive outcomes. The ontology of leader character is such that the dimensions are interrelated and constitute a network of correlated constructs that collectively affect decision-making and action. Hence, we used network-based analysis to appropriately examine the effects of leader character on individually and organizationally relevant variables.

The results of the CFA provide support for a model of 11 intercorrelated leader character dimensions that Crossan et al. identified through qualitative and quantitative research [4]. However, our results also provide a more nuanced picture of leader character. Our CFA revealed the need of modeling a higher-order factor that captured shared variance among the latent and intercorrelated constructs, which we labeled character. Importantly, the results of a network-based analysis revealed that the leader character dimensions and supporting elements are interrelated, forming a connected structure. As hypothesized, judgment emerged as a central dimension in the leader character network. The results also showed that the leader character network was directly connected to follower positive outcomes through drive and judgment. Our third hypothesis was therefore supported as well.

### Theoretical and practical significance

Our study is an important step toward the conceptual development of leader character, which is essential for at least three reasons. First, to fulfill its promise as a foundation for leadership theories: character is an indispensable component of leadership [2, 90]. We view leader character as a critical personal resource that complements or helps to support positive forms of leadership, including, but not limited to, authentic leadership, servant leadership, and ethical leadership. A limitation of these forms of leadership is that by themselves, they fall short in explaining individually and organizationally relevant measures because they represent a rather narrow perspective of what good leadership entails. Put differently, leaders may need to activate multiple leader character dimensions as required by a specific situation to be effective in their role. For example, it is easy to envision how leader character dimensions such as humanity, humility, and collaboration supplement the exercise of authentic leadership because without the ability to activate these dimensions, individuals may perceive the leader as being authentic but in a manner that is dogmatic and abrasive. More specifically, humanity and humility may support dimensions of authenticity to ensure that leaders are transparent and respectful in their relations with followers. Further, courage appears to be essential to exercise authentic leadership. For example, it is never easy and hence requires courage to demonstrate leader behaviors that are guided by internal moral standards and values rather than being based on external pressure such as peers, organizational, and societal pressures.

Second, we extend the structure of the leader character framework and, in particular, establish the central role of judgment, as proposed and reported by Crossan et al. [4]. Fig 1 shows that judgment has its own set of behaviors that underpin it. However, judgment also relies on the ten dimensions that support it. To use a metaphor, judgment is like an air traffic controller: we need leaders who are able to activate each of the 11 dimensions of character at the right time and in the right amount to guide their decision-making and call forth the right behaviors to be successful. Support for the structure of the leader character framework appears to be robust and therefore can serve to redirect empirical research away from approaches that do not honor the interconnectedness of the character dimensions. In addition, both the theoretical framework and methods we propose provide a firm platform from which to conduct subsequent studies as part of a programmatic research effort to examine the antecedents and consequences of leader character at the individual, team, and organizational levels.

Third, our results extend the findings of Crossan et al. [4] by linking the network of leader character dimensions to subjective well-being, resiliency, organizational commitment, and work engagement—critical areas of research in their own right. For example, leader drive connected the character network to follower positive outcomes through affective commitment. Drive can provide the impetus for action. The behaviors associated with drive include striving for excellence, passion, and vitality and remind us that character is not simply a way of thinking but a way of being that may facilitate an emotional attachment to or identification with the organization in followers. At the same time, however, the network structure brings profile to dimensions of character that may have been underestimated, such as temperance. Our experience in assessing and developing leader character in executives reveals that temperance is one of the least developed dimensions in individuals. Temperance relates to the behaviors of being patient, calm, composed, self-controlled, and prudent, and it may play a critical role in whether the person affords themself the opportunity to access the personal resources associated with other character dimensions. We view this as particularly important in situations of adversity and challenge, both in a person’s personal and professional lives. For example, the organization that we studied had experienced significant internal challenges as a result of external factors beyond its control. Temperance to cope with these challenges was therefore a critical personal resource for organizational leaders to have. In sum, our study is the first study of its kind to report a relationship between leader character and individually and organizationally relevant outcomes through examining the topology of a network of relationships. Establishing such links is particularly important since leader character can be developed [1, 23] and hence offers the potential to not only describe the connections between character and individually and organizationally relevant variables but also shift attention to research that helps to prescribe what can be done to strengthen such relations.

The practical significance of our research is at least three-fold. First, we provide clarity as to what leader character is, namely, a habit of being revealed by behaviors anchored in a set of virtues, values, and personality traits. The leader character framework provides a vocabulary for practitioners as it outlines the habits of virtuous behaviors that lead to sustained excellence. Second, we demonstrated the positive implications of leader character on follower functioning. The competency-based perspective—focusing attention on those human resource management activities, functions, and processes that facilitate the development of strategic, organizational, business, and people competencies—remains a dominant force in the field of management. We contend that a shortfall in one of the pillars of good leadership—competencies, character, and commitment—will undermine the other pillars and, eventually, lead to performance problems for leaders, the organizations they lead, and related stakeholders. Therefore, the third practical implication of our research is to consider how to embed leader character in management practices, including selection, performance management, mentoring, and leadership development to cultivate character in current and future leaders. And rather than focusing on only a select few dimensions of leader character, our theorizing and results indicate that organizations should consider the dimensions in a holistic way to fully reap the benefits of character-based leadership.

### Strengths and limitations

Our study has several strengths. First, the study was conducted in a field setting using leader—follower dyads. We sought to understand the relationship between leader character and follower positive outcomes within a single organization as opposed to comparing individual leadership behaviors and subsequent outcomes across samples and contexts. The latter approach would have added myriad contextual variables to our design, potentially attenuating any relationships among leader character and follower positive outcomes. Second, we avoided self-report bias by collecting data from both leaders and followers with a six-month time lag. The time lag helped us to prevent the limitations associated with cross-sectional designs. Third, we used established and validated measures to test our hypotheses. Fourth, we used a novel analytical strategy—namely, network-based analysis—to explore the topology of constructs. This approach allowed us to test the leader character framework developed by Crossan et al. [4] in a robust manner. The technique we used to analyze our data aligns with the ontology of leader character as a set of interconnected dimensions and with the properties of the leader character framework. Further, network-based analysis allowed us to test whether the leader character framework is related to a distinct network of organizationally relevant variables. Fifth, we balanced theory-driven and data-driven approaches. Techniques such as CFA allow researchers to impose relationships among constructs of interest and as such may introduce the potential for researcher bias. It is our belief that an analytical strategy that adequately balances data-driven, exploratory techniques with theory-based, confirmatory modeling brings deeper insights into the research findings than using any method on its own.

A limitation of the study is that we did not construct an experimental setting, nor did we manipulate any variables; hence, we cannot establish causal relationships. For example, we acknowledge that leader character and follower work engagement may be reciprocally related. It is entirely possible that behaviors that reflect work engagement affect the cognitions and behaviors of leaders, including dimensions and elements of their character. For example, leaders may discover that their employees experience the work environment as hostile or non-supportive as a result of leaders’ dedication to advance organizational interests, such as pursuing short-term results that inspire confidence on the part of shareholders. Consequently, work engagement may decline and turnover may increase. This result may encourage leaders to reflect on their behaviors and, subsequently, demonstrate more humanity, justice, and collaboration in future interactions to achieve longer-term results or boost work engagement. The argument that leadership and organizationally relevant outcomes are reciprocally related is consistent with the concept of reciprocal determinism embedded in Bandura’s social cognitive theory [91].

Further, we did not control for contextual factors in our design. Contextual factors such as perceptions of organizational culture, group processes, and economic structures may affect the relationship between leader character and follower positive outcomes. For example, character and work-related attitudes should always be seen in context because what leaders and employees experience is influenced by the presence of others and the environment in which they operate. A third limitation of our study is that we used self-reported assessments of our focal variable, namely, character. Future studies may include reports of peers to validate such self-assessments. The use of self-assessments is common in the behavioral sciences although not ideal because measurement may be affected by memory biases or social desirability.

### Future research

We see several promising areas for future research. First, our results should be replicated and extended to assess the generalizability of the leader character framework. We posit there is an extensive list of organizational processes and outcomes across management disciplines connected to leader character. For example, the notion of leader character is of interest to scholars in organizational behavior (e.g., leadership theories, decision-making, organizational culture, and organizational transformation), human resources (e.g., selection, coaching, and leadership development), and strategy (e.g., strategic leadership, top management teams, diversity, and corporate governance).

Second, causal relationships rather than associations between leader character and individual, team, and organizational outcomes need to be established. For example, researchers should focus on leader character interventions and how the development of leader character affects the way followers evaluate leadership and its consequences. Studies are needed that inform organizational decision-makers how best to develop the leader character dimensions in their employees and leaders so as to improve their personal effectiveness.

Third, we anticipated that judgment would connect leader character to the network of organizationally relevant outcomes. This expectation was supported; however, drive also emerged as a connecting construct. Future studies should continue to investigate in greater detail whether it is judgment that connects leader character to processes and outcomes or if other dimensions also have a critical role to play and, if so, under what circumstances.

Fourth, research designs should incorporate competencies and commitment in addition to leader character in the network since all three have been shown to influence performance [92, 93]. For example, research needs to take into account whether character explains variance in performance over and above competencies and provide evidence of the oft-heard claim that high levels of competencies with weak character is a problem.

## Conclusions

In conclusion, this study serves as an important foundation in the quest to better understand leader character and connect it to many prominent areas of research. The network approach presented enables researchers to connect previously disconnected areas of research, thereby expanding the reach and application of those streams.
